# D- and L-lactate dehydrogenase reactions increase pH to lactate production and decrease pH to pyruvate production

**DOI:** 10.3389/fmolb.2026.1715499

**Published:** 2026-04-07

**Authors:** Robert Robergs, Marek Nalos, Petr Kelbich, Adela Jizerová, Ivana Mervartová, Michal Vostrý, Milan Štengl, Vaclav Babuška

**Affiliations:** 1 School of Exercise and Nutrition Sciences, Faculty of Health, Queensland University of Technology, Kelvin Grove, QLD, Australia; 2 Faculty of Health Studies, Jan Evangelista Purkyne University, Usti nad Labem, Czechia; 3 Department of Physiology, Faculty of Medicine, Charles University, Pilsen, Czechia; 4 Department of Intensive Care Medicine, Pilsen Hospital and Charles University, Pilsen, Czechia; 5 Department of Biomedicine and Laboratory Diagnostics, Faculty of Health Studies, Jan Evangelista Purkyne University, Usti nad Labem, Czechia; 6 Faculty of Environment, Jan Evangelista Purkyne University, Usti nad Labem, Czechia; 7 Department of Medical Chemistry and Biochemistry, Faculty of Medicine, Charles University, Pilsen, Czechia

**Keywords:** acidosis, alkalosis, enzyme catalysis, metabolism, spectrophotometry

## Abstract

**Background:**

The traditional explanation of metabolic acidosis in extrahepatic tissues is the increased cellular production of lactic acid. We tested whether the LDH reaction produces lactate and pyruvate anions, or acids, and measured the pH changes that occur for either direction of the reaction.

**Methods:**

*In-vitro* reagents (25–40 mL) of the D- and L-LDH reaction were prepared for lactate and pyruvate production at known substrate concentrations ([Substrate]), no added H^+^ buffers, at ∼25 °C. The pH of the reagent was measured using a glass polymer pH electrode. The initial reagent pH was adjusted to a target pH value by addition of HCl or KOH (0.02–0.1 N). The baseline pH was recorded for 2 min, followed by the addition of LDH. The pH response was recorded continuously at varied intervals for 20 min. Reagent samples (100–200 μL) were obtained intermittently for measurements of product.

**Results:**

The H^+^ consumption of pyruvate reduction to lactate increased pH (6.3–10.5), and the H^+^ release of lactate oxidation to pyruvate decreased pH (8.8–6.9). The [Product] was small for all [Substrate] and LDH activity conditions (∼0.3 – 0.8 mmol·L^-1^), and lowest for pyruvate production. The pH and [Product] results were similar for both D- and L-LDH reactions. The pH to [Product] profiles for lactate vs. pyruvate equated to 9.215 vs. −9.074 pH units·mmol^-1^·L^-1^, respectively.

**Conclusion:**

Scientists and clinicians are encouraged to use these results to further understand the function of the LDH reaction during perturbations of pH in biological systems.

## 1 Introduction

The enzyme that catalyzes the reduction of pyruvate to lactate, or oxidation of lactate to pyruvate, is lactate dehydrogenase (LDH), which exists in two optical isomer forms; D-LDH and L-LDH. While D-LDH predominates in invertebrates, both LDH optical isomers occur in many bacteria and vertebrates, where L-LDH is the predominant form in mammals (Zewe and Fromm, 1962; Kim et al., 2014; Razeto et al., 2022; Jin et al., 2023) and demonstrates remarkable consistency in the enzyme active site mechanism across the diversity of species (Farhana and Lappin, 2025; Holmes and Goldberg, 2009). That said, there is increasing interest in the D-LDH isomer in humans due to the increased production of D-lactate in specific, though rare, disease conditions (Kim et al., 2014; Razeto et al., 2022; Jin et al., 2023).

The LDH enzyme consists of four domains that can be genetically expressed as either H- (heart) or M- (muscle), where the H-domain predominance increases pyruvate product inhibition (Quistorff and Grunnet, 2011; Maeda et al., 2022). The M-domain has minimal pyruvate product inhibition. H-domain expression predominates in the myocardium and the M-domain is predominantly expressed in skeletal muscle (Zewe and Fromm, 1962; Quistorff and Grunnet, 2011; Eggert et al., 2011) and perhaps certain bacteria (Kim et al., 2014; Razeto et al., 2022). There is also intermediary expression, and as such, the LDH enzyme can occur as H4 (LDH-1), H3M (LDH-2), H2M2 (LDH-3), HM3 (LDH-4), and M4 (LDH-5) (Quistorff and Grunnet, 2011; Maeda et al., 2022). The intermediary forms of LDH occur in tissues such as the liver and kidneys. The LDH domain structure is important, for L-LDH (from rabbit muscle), which was used in this research (Table 1), has product inhibition from both lactate and pyruvate, though the concentration required for meaningful product inhibition is much lower for pyruvate (∼0.2–4.3 mM) than lactate (25–200 mM) (Zewe and Fromm, 1962; Eggert et al., 2011; Wang, 1997; Powers et al., 2007; Latner et al., 1966; Storey, 2016). As previously mentioned, such product inhibition from pyruvate is largest for LDH with greater H-domain expression (Maeda et al., 2022; Eggert et al., 2011; Wang, 1997; Powers et al., 2007; Latner et al., 1966). As will be shown, the comparison of results from D- and L-LDH is interesting in the context of the likely differences in the H- and M-domain expression between the two optical isomers used in this research (Table 1).

**TABLE 1:** Details of the biochemicals used in the pH profiling of the LDH reactions and product assays.

Chemicals	Supplier	Code	Mol weight*
Biochemicals			
β-Nicotinamide adenine dinucleotide sodium salt	Merck	N0632	685.41
β-Nicotinamide adenine dinucleotide, reduced disodium salt hydrate	Merck	N8129	709.40
Sodium D-lactate	Merck	71716	112.06
Sodium L-lactate	Merck	L7022	112.06
Sodium pyruvate	Merck	P5280	110.04
Sodium hydroxide solution (50%)	Merck	415413	40.00
Potassium hydroxide solution (45%)	Merck	417611	56.11
Hydrochloric acid (37%)	Merck	258148	36.46
Hydrazine hydrate solution (24%–26%)	Merck	53847	50.06
Glycine	Merck	G7126	75.07
Enzymes			
D-lactic dehydrogenase from *lactobacillus* leichmannii, lyophilized powder, 1,000 U	Merck	L3888	
L-lactic dehydrogenase from rabbit muscle, lyophilized powder, 5,000 U	Merck	L1254	

* grams·M^-1^.

The reaction mechanism of the LDH enzymes has been widely researched, and despite variations in the multiple transition states during the enzyme catalysis, there remains a dominant sequence of events that support the covalent modifications involved in the reduction of pyruvate to lactate, or the oxidation of lactate to pyruvate, across all LDH isomers and isozymes (Razeto et al., 2022; Jin et al., 2023; Masterson and Schwartz, 2014; Khan et al., 2020). Such events, for the direction to lactate production for human heart LDH, involve pyruvate substrate orientation in the active site by a) ionic interactions between the carboxylic functional group oxygen atoms of pyruvate and the two amine groups of arginine 169, b) ionic NADH interaction with the active site, followed by c) hydride (H^−^; hydrogen with 2 electrons) transfer from NADH (oxidation) to the C2 of pyruvate (reduction), accompanied by d) the joint ionic interaction of the oxygen bound to the pyruvate C2 atom by histidine 193 and arginine 106 (Masterson and Schwartz, 2014). Finally, e) an added H^+^ acquired from the aqueous solution completes the reduction of C2 simultaneous to the charge stability provided by histidine 193 and arginine 106. Minor amino acid sequence differences of the active site between the LDH isozymes result in small variations in the numeric references of the pertinent amino acids, the enzyme catalysis mechanisms and related kinetics (Razeto et al., 2022; Farhana and Lappin, 2025; Masterson and Schwartz, 2014; Khan et al., 2020; Read et al., 2001).

Pyruvate+NADH+H+ ⟔L−LDHLactateL+NAD+ (1)

Pyruvate+NADH+H+ ↔D−LDHLactateD+NAD+ (2)

Regardless of the species differences in D- vs. L-LDH expression, as previously explained, D-LDH and L-LDH catalyze the same reaction, except for the specificity of the D- and L- lactate optical isomers (Equations 1, 2). The substrates are the same in the direction of D- or L-lactate production, and the products are the same in the direction of pyruvate and NADH production. In other words, for the sake of this research, the hydrogen ion (H^+^) involvement in a chemical reaction, or what we now refer to as H^+^ exchange (H^+^_e_) is the same whether as a substrate for D- or L-lactate, or an added product of pyruvate production, depending on the reaction direction. Consequently, we learn the same information about the H^+^_e_ of the LDH chemical reaction, regardless of whether the reaction is catalyzed by D- or L-LDH. This has importance for the purpose of this research and the related data collection and interpretation.

There is biochemical, physiological and clinical interest in the D- and L-LDH reaction for many reasons. There has been a long exercise physiology interest in skeletal muscle metabolic acidosis during sustained, repetitive, intense muscle contractions. For much of this history since the Nobel Laureate research of Hill and Meyerhoff (Raju, 1998; Hill and Lupton, 1923), explanation of the exercise-induced metabolic acidosis has been assumed to be caused by the cellular production of L-lactic acid (Robergs et al., 2004; Robergs, 2017; Robergs, 2019; Robergs, 2021; Torrens et al., 2023; Sehelt et al., 2017; Kreisberg, 1980; Katz and Sahlin, 1988; Voet and Voet, 2018; Nelson and Cox, 2021; Robergs, 2001). The interpretation was that the H^+^ release from lactic acid during pH dependent H^+^ dissociation contributes to the development of metabolic acidosis. The added historical events that led to the theory that cells produce metabolic acids has been explained elsewhere (Lesney, 2003; Robergs et al., 2004).

Clinically, cellular metabolic acid production has also been the dominant theory for explaining the systemic (blood) acidosis of numerous disease processes. The hyperlactatemia and acidosis (L-lactic acidosis) that accompanies critical illnesses like trauma, sepsis, the ingestion or inhalation of toxins, and circulatory shock are associated with significant increases in incidence (Lee et al., 2008) and odds ratios for in-hospital mortality (Wernly et al., 2020). The cellular metabolic acidosis theory is also core to the current interpretation and treatment of the acidosis that accompanies severe ketosis (keto-acidosis), such as during starvation or poorly controlled diabetes (Torrens et al., 2023; Dhatariya et al., 2020). More recently, research of all D- and L-LDH enzymes has gained added importance due to the enhanced rates of glycolysis in cancers, revealing possible strategies for inhibition of LDH and as such the imposition of major energetic constraints to the ATP provision of glycolysis and growth of cancerous tissue (Kim et al., 2014; Razeto et al., 2022; Jin et al., 2023; Farhana and Lappin, 2025; Maeda et al., 2022; Khan et al., 2020; Chen et al., 2024; Gray et al., 2014). Interestingly, as of 2024, clinical researchers still refer to cellular metabolism in the context of lactic acid and pyruvic acid production (Chen et al., 2024).

The lactic acid theory was opposed as early as 1977 (Gevers, 1977), with further supporting interpretations published through to 1991 (Zilva, 1978; Wilkie, 1979; Dennis et al., 1991). Such research and commentary proposed that cells produced lactate and the likely cause of the H^+^ release causing acidosis was ATP hydrolysis. In 1983 Peter Stewart proposed that metabolic acids to not cause systemic acidosis (Stewart, 1983). Rather, Stewart presented theoretical evidence for the role of adjustments in strong ions and the arterial partial pressure of carbon dioxide (PaCO_2_) to cause alterations in the pH of biological solutions. This approach, known as the Physico-Chemical Theory, has been influential in explaining pH changes induced by perturbations in the concentrations of cations and anions in blood and as such has attracted moderate clinical support (Po and Senozan, 2001; Sirker et al., 2012; Aiken, 2013; Paik, 2015; Morgan, 2009). Nevertheless, there has been unconvincing evidence from attempts at validation of this approach from human blood samples (Kowalchuk and Scheuermann, 1994). Furthermore, Stewart was clear in how the physico-chemical approach assumed, though no evidence in support of this view was presented, that there were no direct changes to the pH of biological solutions resulting from chemical reaction H^+^_e_ via metabolic release or consumption; “*…. since [H* ^*+*^ *] is a dependent variable, H* ^*+*^ *movements into or out of a solution do not provide quantitative explanations for changes in [H* ^*+*^ *].*” (Stewart, 1983) (page 1445).

Evidence from computational chemistry application of dissociation constants to understand the H^+^_e_ from chemical reactions has repeatedly shown that cells produce lactate not lactic acid, and that some chemical reactions that involve H^+^ as a substrate or product may influence the pH of the aqueous environment they are in (Robergs, 2017; Robergs, 2019; Robergs, 2021; Kushmerick, 1997; Vinnakota et al., 2006). Further application of this approach to the modelling of skeletal muscle energy metabolism during intense muscle contractions estimated a gross H^+^ release to lactate production stoichiometry of > 4:1 (Robergs, 2017; Robergs, 2019; Robergs, 2021).

The reading of any biochemistry text reveals a major contradiction in the content presented for the structures of many of the metabolites of cellular energy catabolism and their supported text explanations of the cause of metabolic acidosis. Many of the substrates and products of a subset of these reactions contain a carboxylic acid functional group, and are therefore referred to as carboxylic acids, even though they are produced in their ionic form (devoid of a hydrogen in the carboxyl group), and thereby do not, and cannot release a H^+^ into solution. The cellular production of lactic acid, or lactate, is a classic example of such a carboxylic acid. The biochemistry textbooks provide structural chemical evidence that oppose the cellular production of metabolic acids; as previously stated, the acid metabolites are produced in their ionic (base) form. However, comments are then made to reinforce the cellular metabolic acid construct, predominantly using lactic acid as an example, such as, “*… the meat of an animal that has run to exhaustion before being killed has a sour taste. This is a result of lactic acid build-up in the muscles.*” (Voet and Voet, 2018) (page 631); “*Since ketone bodies are acids, their high concentration puts a strain on the buffering capacity of the blood …*” (Voet and Voet, 2018) (page 978); “*…. Organic nutrient molecules (carbohydrates, fats and proteins) are converted to smaller, simpler end products (such as lactic acid, CO*_*2*_ *and NH*_*3*_ *).*” (Nelson and Cox, 2021) (p. 1783); “*… the acidification that results from ionization of lactic acid in muscle and blood limits the period of vigorous activity.*” (Nelson and Cox, 2021) (p. 2031).

**FIGURE 1:**
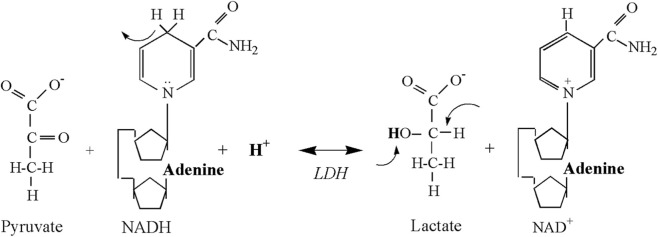
The chemical structures of the substrates and products, and their atomic rearrangements, during the lactate dehydrogenase reaction. Re-used Figure with approval from Robergs et al. (2004).

Then there is evidence of the LDH reaction itself (Figure 1), where the production of lactate consumes close to 1 H^+^ per lactate molecule across the cellular (skeletal muscle) physiological pH range (6.0–7.0) (Robergs et al., 2004; Robergs, 2017; Robergs, 2019; Robergs, 2021; Torrens et al., 2023; Kushmerick, 1997; Vinnakota et al., 2006). When applying an understanding of organic chemistry, an evidence-based hypothesis would be that this reaction should raise, not lower, the pH of biological solutions. Such evidence leads to the obvious question; if chemical reactions within cells do not produce acid molecules, then where do the H^+^ come from that cause cellular and/or systemic metabolic acidosis?

Consequently, as of 2025, there remains a diverse account, across multiple theories, for the cause of metabolic acidosis. A reasonable synopsis of this scenario leads to the conclusion that there is no dominant theoretical or evidence-based understanding of the causes (there is no logic in assuming there is only one cause) of metabolic acidosis at the cellular or systemic (blood) level. Based on Kuhn’s (1962) account of the historical development of the scientific achievements across the physical science disciplines, such a scenario of numerous documented anomalies and competing theories of the cause of metabolic acidosis amount to a state of ‘crisis’ for the scientists and practitioners of acid-base chemistry and physiology. Similar crises would also be experienced by the additional disciplines and professions influenced by acid-base chemistry and physiology, such as exercise and sport sciences, and medicine.

Given the importance of the differentiation between lactic acid and lactate production to cellular energy transfer, chemical reaction H^+^_e_, and the understanding of the cause of acidosis, the purpose of this research was to pursue exploratory analyses of the changes (amount and direction) in the pH of an *in-vitro* reagent without added H^+^ buffering during the catalysis of each of the D- and L-LDH reactions. Research inquiry focused on both directions (lactate vs. pyruvate production) of the reactions at different enzyme activity and substrate concentrations ([S]). Such results would directly answer or confirm; 1. whether the LDH reaction, depending on direction, produces lactate and pyruvate as opposed to lactic acid and pyruvic acid; 2. whether the H^+^_e_ as either a substrate or a product of a chemical reaction directly alters the pH of the *in-vitro* reagent of the chemical reaction; 3. whether the H^+^_e_ and resulting pH profiles are consistent with the organic chemistry of the LDH reaction and are therefore similar for D- and L-LDH, 4. does the pH profile of the H^+^_e_ of the LDH reaction support the prior computational chemistry evidence? does the resulting [Product] (x-axis) to [H^+^] (or H_3_O^+^ activity; _a_H_3_O^+^) or pH profile (y-axis) for both directions of the LDH reaction, regardless of the optical isomer, reveal a computational strategy for documenting the direct influence of the H^+^_e_ of chemical reactions on the resulting pH of the *in-vitro* reagent.

As a chemical reaction in an *in-vitro* reagent functions similarly (the same substrates, products and resultant atomic rearrangements) as in a cell, such data could provide experimental evidence for novel, or refined understanding of the cellular and systemic acid-base chemistry that may apply to biological systems. It is only when the basic science understanding of the H^+^_e_ of chemical reactions, and how such involvement does or does not directly influence pH is understood, can a collective of evidence be used to best account for what is likely to be a multifaceted and condition specific cause of metabolic acidosis within more complex biological systems.

## 2 Methods

The details of the chemicals used for the study of the LDH reaction are presented in Table 1. As shown, all chemicals and enzymes used in this study were purchased from MERK KGaA (Darmsadt, Germany). The [S], reaction direction, main product peak concentration ([Product]), and enzyme activity used for the different reaction conditions for quantifying the pH changes for lactate vs. pyruvate production are presented in Tables 2, 4.

**TABLE 2:** Details of the biochemicals used in the pH profiling of the LDH reactions and the pH conditions of the substrate reagent before and after adjustment to the target start pH.

#	Substrate*	→ Product	Amount (g)	mM⋅L^-1^^	Enzyme (U)	Vol_start_ (mL)^^^	pH_i_	pH_start_^#^	pH_end_	[Prod] ^δ^ (mM⋅L^-1^)
LL1	Na^+^-Pyruvate	Lactate	0.0149	3.31	L-LDH:5	38.9	7.45	6.18	9.78	
	Na^+^-NADH		0.0150	0.94						
LL2	Na^+^-Pyruvate	Lactate	0.030	10.91	L-LDH:10	24.06	7.33	6.22	9.87	0.4
	Na^+^-NADH		0.1728	9.74						
LL3	Na^+^-Pyruvate	Lactate	0.0158	5.74	L-LDH:50	25.02	6.84	6.45	10.31	
	Na^+^-NADH		0.0531	3.0						
LL4	Na^+^-Pyruvate	Lactate	0.0141	5.12	L-LDH:10	25.01	6.84	6.56	10.09	
	Na^+^-NADH		0.0536	3.02						
LL5	Na^+^-Pyruvate	Lactate	0.0085	3.09	L-LDH:5	25.0	6.48	6.50	9.87	0.2
	Na^+^-NADH		0.0538	3.03						
LL6^tr^	Na^+^-Pyruvate	Lactate	0.0093	3.36	L-LDH:20	25.03	7.67	6.30	9.95	
	Na^+^-NADH		0.0543	3.06						
LL7^tr^	Na^+^-Pyruvate	Lactate	0.0090	3.25	L-LDH:20	25.03	7.41	6.21	10.01	
	Na^+^-NADH		0.0570	3.21						
DL1	Na^+^-Pyruvate	Lactate	0.1190	26.68	D-LDH:25	39.08	6.98	6.65	10.8	0.8
	Na^+^-NADH		0.2021	7.05						
DL2	Na^+^-Pyruvate	Lactate	0.0423	9.85	D-LDH:10	39.50	7.43	6.9	10.55	
	Na^+^-NADH		0.1747	6.31						
DL3	Na^+^-Pyruvate	Lactate	0.0221	5.07	D-LDH:10	39.06	7.81	6.26	10.54	
	Na^+^-NADH		0.0658	2.30						
DL4	Na^+^-Pyruvate	Lactate	0.0137	3.11	D-LDH:1	39.0	7.18	7.13	9.93	
	Na^+^-NADH		0.0669	2.15						
DL5	Na^+^-Pyruvate	Lactate	0.0221	5.07	D-LDH:20	39.08	7.06	6.4	9.94	
	Na^+^-NADH		0.0658	2.30						
DL6^tr^	Na^+^-Pyruvate	Lactate	0.0090	3.25	D-LDH:5	25.0	7.02	6.92	10.13	
	Na^+^-NADH		0.0538	3.03						
DL7^tr^	Na^+^-Pyruvate	Lactate	0.0090	3.25	D-LDH:5	25.0	6.95	6.97	10.16	
	Na^+^-NADH		0.0543	3.06						
LP1	Na^+^-L-Lactate	Pyruvate	0.0230	5.13	L-LDH:10	39.3	5.7	9.38	7.98	0.06
	Na^+^-NAD^+^		0.1361	5.13						
LP2	Na^+^-L-Lactate	Pyruvate	0.0179	6.52	L-LDH:50	24.51	5.56	8.0	7.55	
	Na^+^-NAD^+^		0.0850	5.06						
LP3	Na^+^-L-Lactate	Pyruvate	0.0095	3.50	L-LDH:10	24.19	5.8	9.15	8.15	
	Na^+^-NAD^+^		0.0510	3.08						
LP4	Na^+^-L-Lactate	Pyruvate	0.0084	2.93	L-LDH:20	24.34	5.8	8.26	8.05	
	Na^+^-NAD^+^		0.0514	3.04						
LP5^tr^	Na^+^-L-Lactate	Pyruvate	0.0087	3.03	L-LDH:60	24.1	5.73	7.96	7.76	
	Na^+^-NAD^+^		0.0528	3.08						
LP6^tr^	Na^+^-L-Lactate	Pyruvate	0.0085	2.97	L-LDH:60	24.11	5.81	7.90	7.59	
	Na^+^-NAD^+^		0.0521	3.01						
DP1	Na^+^-D-Lactate	Pyruvate	0.1105	24.16	D-LDH:10	39.81	5.76	8.76	6.72	0.3
	Na^+^-NAD^+^		0.5312	19.62						
DP2	Na^+^-D-Lactate	Pyruvate	0.0222	4.84	D-LDH:10	40.1	5.75	8.31	6.89	0.1
	Na^+^-NAD^+^		0.1342	4.95						
DP3	Na^+^-D-Lactate	Pyruvate	0.0141	3.10	D-LDH:0.1	40.53	5.68	8.47	7.24	
	Na^+^-NAD^+^		0.0797	2.96						
DP4^tr^	Na^+^-D-Lactate	Pyruvate	0.0085	3.03	D-LDH:5	24.09	5.62	7.99	7.31	
	Na^+^-NAD^+^		0.0529	3.09						
DP5^tr^	Na^+^-D-Lactate	Pyruvate	0.009	3.21	D-LDH:5	27.08	6.09	7.92	7.30	
	Na^+^-NAD^+^		0.052	3.04						

*Including added needed chemicals (see Methods); ^adjusted for acid/base addition; ^#^pH_start_ = pH at the start of the reaction, prior to the addition of the enzyme; pH_end_ = pH at 20 min; ^δ^from test tube assays; ^tr^used for test re-test reliability.

The methods of this research can be divided into eight phases. The first phase involved the calibration or checking of the pH meter (pH50 Lab pH Meter, Westlab, Ballarat Victoria, Australia) using known pH calibration standards at pH = 4.0, 7.0 and 10.0. The pH electrode was a maintenance free, open pore, Ag/AgCl glass polymer electrode with an integrated temperature sensor (CHS Polymer Green Temp (NTC30), Code CHS-5670-T1B; Chromservis, Petrovice, Czech Republic). If pH values were within ± 0.03 units for each of the three pH standards, no calibration was performed. If one reading was in error by more than this amount, a new calibration was performed. Typically, an acceptable calibration was retained for at least 4 days. The calibrated pH electrode was placed into a sealed plastic pH electrode tube containing a salt solution (2 mL sample of 30 g KCl in 100 mL) until the next method phase was completed, in addition to all times the pH meter was not used.

The second phase was the preparation of the chemical reaction reagent for the direct measurement of pH as detailed in Table 2, in addition to the measurement of the pH of deionized water so that the reagent initial pH (pH_i_) could be better interpreted. Reactions were completed for both the D- and L-LDH optical isomers, though note from Table 1 that the D-LDH and L-LDH were from different biological species and as such likely to have different H- to M-domain expression and resultant proportionality, different extents of pyruvate and lactate inhibition, and different kinetics. The weights required of all chemicals were calculated based on the supplier’s stated molecular weights, and expression of [Substrates] (mmol·L^-1^) were adjusted for reagent volume (39 or 24 mL + the volume of added hydrochloric acid (0.1 N HCl) or potassium hydroxide (0.1 M KOH) to attain targeted pH (_start_pH) values. All reagents were prepared using deionized water (pH = 5.08–6.22), along with any enzyme activators, substrate stabilizers, and of course, [S] for different reagent conditions. For each chemical reaction, the initial [Substrates] ranged from 0.94 to 26.7 mmol⋅L^-1^, the [Products] were always 0.0 mmol⋅L^-1^, and enzyme activities (total U for the reagent volumes) ranged from 0.1 to 60 International Units (U) (Table 2). Note from Table 1 that all enzymes were lyophilized preparations from biological tissue, with no added H^+^ buffers. Dilution of the enzyme to suit needed activities for specific reaction conditions was completed with deionized water.

Once the reagent was made, phase 3 occurred, where the reagent beaker was placed on a heated magnetic stirrer to attain and retain a temperature as close as possible to 25 °C (standard temperature in chemical analyses), and the pH electrode was placed in the beaker at a depth sufficient to ensure adequate electrode tip performance without being impacted by the magnetic stirrer bar. The magnetic stirrer was set to cause a rod rotation at close to 4 Hz, though this rate was mildly variable (±2 Hz) across the duration of the reaction and data collection.

Once the reagent pH stabilized, the value was recorded as the initial pH reading of the reagent. As such, and as previously explained, the total reagent volumes were approximately 40 mL, and for latter pH profiles 25 mL, with slight modifications to the final volume depending on the exact volume of acid or base (0.1, or 0.02 N HCl or K^+^OH^−^) required to have the reagent attain the targeted _start_pH values. These desired pH values differed depending on the expected pH change and direction for each LDH reaction direction (Robergs, 2017; Robergs, 2019; Robergs, 2021; Kushmerick, 1997; Vinnakota et al., 2006). For example, given the H^+^ consumption of the LDH reaction to lactate, the _start_pH condition was approximately 6.5, whereas the H^+^ release that occurs for LDH to pyruvate, the _start_pH was approximately 8.5 (Table 2). There was variability in attaining _start_pH values based on the pH sensitivity of a reagent without added H^+^ buffers. The added acid or base volumes were recorded and once a stable pH was established, the adjusted pH value was recorded. All presented [Substrates] of Table 2 are adjusted for final reagent volumes, as previously described.

Phase 4 involved the set-up of the video recording of the reaction, and a stopwatch placed next to the pH meter. The video recording was then started, and the chemical reaction commenced by the addition of the required volume of known standard enzyme solution (typically 10–60 μL) equating to 0.1–50 U, depending on the enzyme. The stopwatch was then started (<2 s delay), and the pH readings were manually recorded as follows: every 10 s from 0 to 2 min, every 20 s from 2 to 5 min, every 30 s from 5 to 10 min, then every 1 min to 20 min. For most reactions, 100 μL samples of the reagent were sampled and dispensed into 100 μL of 0.1 N HCl in pre-labelled (numerically) 1.5 mL sample vials for a distributed subset of the pH time conditions. For latter reactions, this sample volume was doubled (200 μL) to improve the sensitivity of product measurement from spectrophotometry. The timing and frequency of the sampling depended on the specific chemical reaction, but most samples were, or very similar to being baseline, then every 10 s for 1 min, then every 20 s for 1 min, then every 1 min for 4 min, then every 2 min for 4 min, then every 3 min for 10 min. Such data recording either involved 2 people (sampling vs. pH recording), or one person (sampling throughout, but no pH recording manually until after 2 min where both tasks could then be completed by 1 person). The initial 2 min of pH values were observed from the video recording.

**FIGURE 2:**
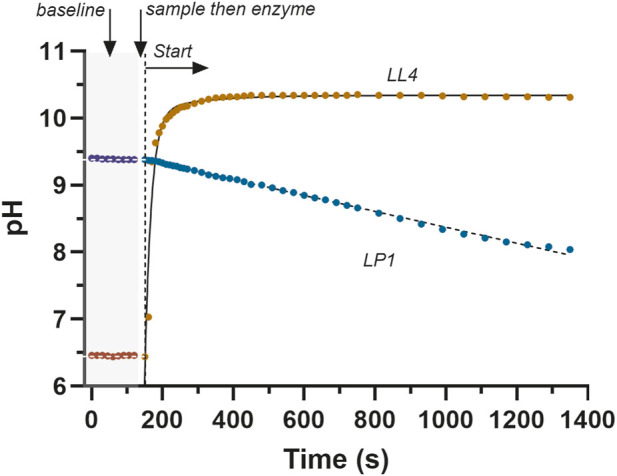
Select data from the reactions of Table 2 (LL4, LP1, see Table 2) that document the stable baseline pH of the L-LDH reaction in the directions of lactate and pyruvate production. The stable baseline was consistent across all LDH isozyme, product direction conditions and the range of pH values covered in this research.

To document the stability of the _start_pH values, for a subset of the LDH reaction pH profile reactions the pH of the deionized water was first quantified. Once the reagent was prepared and pH corrected to the targeted _start_pH value, the reagent pH was measured every 15 s during a baseline data collection phase of 2 min. For these trials, once this was completed, the timer was reset, a baseline sample of the reagent was obtained, and the enzyme was then added as previously detailed, followed by the start of the timer once again. The timing of these events for representative conditions of the L-LDH reaction for each of lactate and pyruvate production are presented in Figure 2.

Phase 5 occurred after the completion of the 20 min data recording of the pH changes during the chemical reaction. All sample vials were then mixed by electrical/mechanical vortex. The batch of samples was then labelled (reaction and date) and placed in freezer storage (−20 °C) until subsequent assay via NAD^+^-NADH coupled enzyme spectrophotometry (phase 6).

This procedure was also performed for specific reaction conditions (start pH, enzyme activities and [Substrate]) for repeat acquisitions of the pH profiles of the H^+^_e_ of the LDH reactions for each LDH isozyme (L-LDH vs. D-LDH) for both directions (lactate vs. pyruvate production) of the reactions. Such procedures were performed to quantify the test re-test reliability (correlation coefficient) and validity (residuals error) of the method and pH responses of the H^+^_e_ of the chemical reactions. For these reactions, each reaction condition involved the preparation of a new reagent. This was done to include all possible method error, in addition to chemical reaction error, into the test re-test assessment. Due to the differences in LDH isozyme catalytic activity and regulation from pyruvate and lactate, different enzyme activities were used for the L-LDH vs. D-LDH (60 vs. 5 U, respectively; Table 2; see LL6^tr^, LL7^tr^, DL6^tr^, DL7^tr^, LP5^tr^, LP6^tr^, DP4^tr^, DP5^tr^). For each replicate reaction, the same enzyme activity was used, and [Substrate] and start-pH values were prepared as close as possible to each other (small error remained due to the need to modify the _start_pH for the direction to pyruvate production (this is difficult in an unbuffered reagent), and for the metabolite sample weights).

Phase 6 utilized two different methods of spectrophotometry (Carey-60 UV-Vis, Agilent, Santa Clara, California, United States). For the first and traditional method, the frozen samples obtained from the reaction pH profile methods were thawed to room temperature, vortex mixed, and then 25 μL (for later assays this increased to 70–100 μL) was added to 1 mL of a reagent in borosilicate test tubes. For the lactate assay, the reagent consisted of 32 mmol·L^-1^ glycine, 2 mmol·L^-1^ hydrazine hydrate, 1 mmol·L^-1^ NAD^+^, in deionized water, with 2 U·mL^-1^ using D-LDH or 5 U·mL^-1^ using L-LDH, though was later modified by halving the glycine and hydrazine concentrations to improve stability of the spectrophotometer absorbance measures. Tubes were capped, vortex mixed, returned to their tube rack and then incubated at 35 °C in a heated water bath for 40 min. Each sample was manually transferred to a 12.5 × 12.5 × 40 mm (10 mm path length) quartz crystal cuvette and lactate production was measured against a baseline blank in duplicate as the increase in absorbance (pyruvate production in the assay) at 340 nm. For pyruvate production, the assay was initially based on measuring the decrease in lactate using the same regent of the lactate assay. However, such a method proved to be too insensitive to small changes in lactate, most likely due to the energetically unfavorable direction of the LDH reaction. Consequently, pyruvate was subsequently assayed as the decrease in absorbance as pyruvate is converted to lactate based on the reagent assay conditions of 12.5 mmol·L^-1^ Tris base, 5 mmol·L^-1^ HCl, 0.2 mmol·L^-1^ NADH, in deionized water, with 2 U·mL^-1^ using D-LDH or 5 U·mL^-1^ using L-LDH.

The cuvette method involved repeating the LDH reaction of the pH profiling condition (best efforts were pursued to mimic the [Substrates] and enzyme activity, in addition to added combinations) in a 1 mL sample volume within a quartz crystal cuvette (Hellma Analytics, 12.5 × 40 mm, 1.5 mL volume capacity, Hanover, Germany) within the spectrophotometer (Table 3). The method differed from the pH profiling for pyruvate production from the LDH reaction due to the need to add hydrazine to destroy/remove the pyruvate and thereby prevent product inhibition of the LDH, as well as provide H^+^ buffering in addition to glycine. This procedure was straight forward for the LDH reaction to pyruvate, as baseline reagent conditions involved NAD^+^ and not NADH. Based on manufacturer data, the functional absorbance range of the spectrophotometer was 0–4 units. For NADH, based on Beer’s Law, 4 absorbance units equates to 0.643 mmol·L^-1^. As will be presented in Results, the resulting pyruvate production mostly remained < 0.6 mmol·L^-1^. For the LDH reaction to lactate, the need for baseline NADH in the reagent, and the measurement of the decrease in NADH absorbance at 340 nm prevented the absorbance measurement for most of the reaction due to being far in excess of the absorbance range of the spectrophotometer. Consequently, for lactate production, these direct spectrophotometer reactions used the difference between the calculated baseline NADH and the NADH for the absorbance data < 3.0 units (data between 3 and 4 absorbance units remained too unreliable) to calculate lactate production. Due to the highly exergonic nature of the LDH reaction to lactate production, it was expected that lactate production via this cuvette method would be large and in near completion of the total baseline NADH. Such methods and example results are presented in Figures 5a,b.

**TABLE 3:** Data for the test re-test reliability of the chemical reactions for both L-LDH and D-LDH, and each reaction direction to lactate vs. pyruvate.

Variable	L-LDH to La	L-LDH to Pyr	D-LDH to La	D-LDH to Pyr
n=	29	38	39	38
Slope 95% CI=	1.061 to 1.110	0.8067 to 0.8987	0.9060 to 0.9627	0.9042 to 0.9349
R^2^=	0.9980	0.9766	0.9918	0.9976
S_y.x_=	0.0240	0.0104	0.0206	0.009
F=	22872	1419	4462	14765
df=	1:27	1:36	1:37	1:368
p=	<0.0001	<0.0001	<0.0001	<0.0001
Equation	(1.080 * x) – 0.8700	(0.8527 * x) + 1.106	(0.9343 * x) + 0.7207	(0.9195 * x) + 0.5414

La = lactate; Pyr = pyruvate; n = data points per sample used in the linear regression; S_y.x_ = standard error of the estimate for the pH data of each condition used in the linear regression; Equation = final regression equations ((slope * x) ± y-intercept).

The standard reagent for lactate production consisted of 5 mmol·L^-1^ Na^+^Pyruvate, 5.0 mmol·L^-1^ Na^+^NADH hydrate, 16 mmol·L^-1^ glycine, in deionized water, with 0.1 U·mL^-1^ using D-LDH and 1 U·mL^-1^ using L-LDH, totaling 5 mL, with specific modifications of [S], enzyme isozyme, and enzyme activity as needed. The standard reagent for pyruvate production consisted of 5 mmol·L^-1^ Na^+^Lactate, 5.0 mmol·L^-1^ Na^+^NAD^+^, 32 (initially, then this was lowered) to 16 mmol·L^-1^ glycine, 2 (initially, then this was lowered) to 1 mmol·L^-1^ hydrazine, in deionized water, totaling 5 mL, with specific modifications of the lactate optical isomer, [Substrates], and enzyme activity as needed. As previously explained, the cuvette method was only pursued to further document the successful catalysis (product formation) of the chemical reaction in the intended direction, with further verification of the approximate extent of maximal product formation during the pH profiling (from the direction of pyruvate production as previously explained).

For comparison of measured pH and [Product] data to computed expected pH changes for H^+^ addition to water based on a modified Henderson-Hasselbach equations, the following methods were performed. To develop a computational approach at understanding and predicting the pH changes for chemical reaction H^+^_e_, it was best to start with the Henderson-Hasselbach equation (Equation 3) and then modify it as needed based on a reverse derivation (Po and Senozan, 2001). This explanation commences with the LDH reaction in the direction of pyruvate production, and as such, H^+^ release (−‘ve H^+^_e_), and then presents the calculations for the direction of lactate production. Based on convention, the [H^+^] is used to express the increased _a_H_3_O^+^, which is involved in near instantaneous repeated H^+^_e_ with neighboring water molecules, and where this activity increases with increasing H^+^ release in an aqueous solution.

$$pH=pK_{a}+\log_{10}\left(\frac{\left[A^{-}\right]}{\left[AH\right]}\right)$$ (3)

where $pK_{a}$ = −log of the dissociation constant; $\left[A^{-}\right]$ = concentration of the base; $\left[HA\right]$ = concentration of the acid

As the H^+^_e_ of a chemical reaction that releases a H^+^ (–’ve H^+^_e_) (LDH reaction to pyruvate), or consumes a H^+^ (+’ve H^+^_e_) (LDH reaction to lactate), does not have a dissociation constant given the H^+^ release is total and pH independent, there is no pKa component required. As such there is also no need for a ratio of the ionic to acid form of the acid metabolite as there is no acid metabolite as the source of the H^+^. Therefore, for the LDH reaction in the direction of pyruvate production and a –’ve H^+^_e_, the [P] replaces the [HA] of the Henderson-Hasselbach equation (Equation 1). Correcting for the -log moves the [P] component to the numerator as shown in Equation 4. A [P] = 0.001 M is used to exemplify the computations. At this stage, it is important to emphasize that in providing these modified equations, we are not proposing that they are correct. We are simply projecting a computational strategy into a new realm of inquiry and knowledge as a start to ascertaining the relevance, or not, of this approach.

$$pH=-\log\left[Product\right]$$ (4)

$$pH=-\log_{10}0.001=3$$

In this account, the product formation is directly stoichiometric to the pH and therefore the [H^+^], which now equates to the chemical reaction, not an acid metabolite, as the source of the H^+^. As will be exemplified later, Equation 4 must be expressed differently for the H^+^ consumption (+’ve H^+^_e_) of the LDH reaction to lactate production.

The pH can now be converted to the [H^+^] (Equation 5).

$$10^{-pH}=\left[H^{+}\right]$$ (5)

$${\left[H^{+}\right]=10}^{-3}=0.001\;M$$

Given that water is the source of some H^+^ involved in cellular metabolic H^+^_e_, and the destination of other H^+^ involved in chemical reactions that also contribute the H^+^_e_, it is pertinent to now include the dissociation constant of water (K_w_ = 1.0 × 10^−14^) into this equation, which first involves the calculation of the [OH^−^] (Equations 6,7 [Disp-formula e7]).

As the product of [H^+^] and [OH^−^] equals 10^−14^ (Equation 6), the [OH^−^] is easy to calculate when the [H^+^] is known (Equation 7). This step is important for the calculations involving the correction for the ionization of water in solutions with meaningful ionic strength ( I ) as explained below.

$$10^{-14}=\left[H^{+}\right]\times \left[{OH}^{-}\right]$$ (6)

$$10^{-14}=\left[0.001\right]\times \left[{OH}^{-}\right]$$

$$\left[{OH}^{-}\right]=\frac{10^{-14}}{\left[0.001\right]}=1.0\;x\;10^{-11}\;M$$ (7)

To adjust the pH by the ionization constant of water (K_w_ = 1.0 × 10^−14^) (assumes T = 25 °C and I = 0), the presence of ions in the reagent solution ( I varies with [Substrates], which for this research [Substrates] was between ∼ 2 and 25 mmol⋅L^-1^) requires that this constant be adjusted for ionic strength (Equation 8). Based on the data of Harned and Owen (1958) and given the LDH reaction to pyruvate and to lactate had ionic strength values that were all small and close to approximately 0.03 M, the Kw has been adjusted slightly (K_w_ = 1.0 × 10^−13.8^) for a more accurate account of the following computations.

$$I=\frac{1}{2}\sum_{i=1}^{n}b_{i}z_{i}^{2}$$ (8)

where I = ionic strength (M), b = [ion] (M·L^-1^), and z = charge number of the ion

The altered Kw ( ${iK}_{w}$ ) can now be used to compute the altered [H^+^] based on Equation 9.

$${\left[H^{+}\right]}_{adj}=\frac{{iK}_{w}}{\left[{OH}^{-}\right]}$$ (9)

For LDH to Pyruvate;

$${\left[H^{+}\right]}_{adj}=\frac{1\times 10^{-13.8}}{1\times 10^{-11}}$$

=0.0015849 M

Compared to the results of Equation 5, the ionic strength adjustment of the Kw induces a meaningful increase in H^+^ release.

For the LDH reaction to lactate production and a +’ve H^+^_e_, Equation 2 must be re-expressed to Equation 10 to adjust the pH expression resulting from H^+^ release to provide the proportional change in pH resulting from H^+^ consumption.

$$pH=7-\left(-\log_{10}0.001\right)+7$$ (10)

pH=7−3+7=11

The following calculations can then be made based on the preceding explanations and equations.

$${\left[H^{+}\right]=1\times 10}^{-11}\;M$$

$$\left[{OH}^{-}\right]=\frac{10^{-14}}{\left[10^{-11}\right]}=0.001\;M$$

$${\left[H^{+}\right]}_{adj}=\frac{{iK}_{w}}{\left[{OH}^{-}\right]}$$

For LDH to lactate;

$${\left[H^{+}\right]}_{adj}=\frac{1\times 10^{-13.8}}{1\times 10^{-3}}$$

$$\;=1.5849\times 10^{-11}\;M$$

### 2.1 Data processing and statistics

All data were entered into a commercial spreadsheet program (Excel, Microsoft Corporation, Redmond, WA, United States), where pH data were converted to [H^+^] expressed as M × 10^−9^. Such data were then imported into a commercial curve fitting and graphics program (Prism, v10.6.1 (892), 2025, GraphPad Software, Boston, MA, United States). All pH-time profiles and product formation-time profiles were processed for best fit (lowest standard error of the estimate; SE) linear or non-linear functions. The equations resulting from the best fit linear or non-linear functions were then used to graph pH vs. [Product] data in addition to [H^+^] vs. [Product] data.

To derive data for the pH vs. [Product] profiles, the non-linear equations and their resulting coefficients for pH vs. time and [P] vs. time were programmed into custom software (LabVIEW™, v-2017, National Instruments, Austin, TX, United States) and data were generated at 1 Hz across the 20 min of each reaction data collection, providing 1,201 data points for each profile. These results were compiled into two-dimensional arrays and then saved as.txt files for subsequent importing into the commercial curve fitting and graphics program.

**FIGURE 3:**
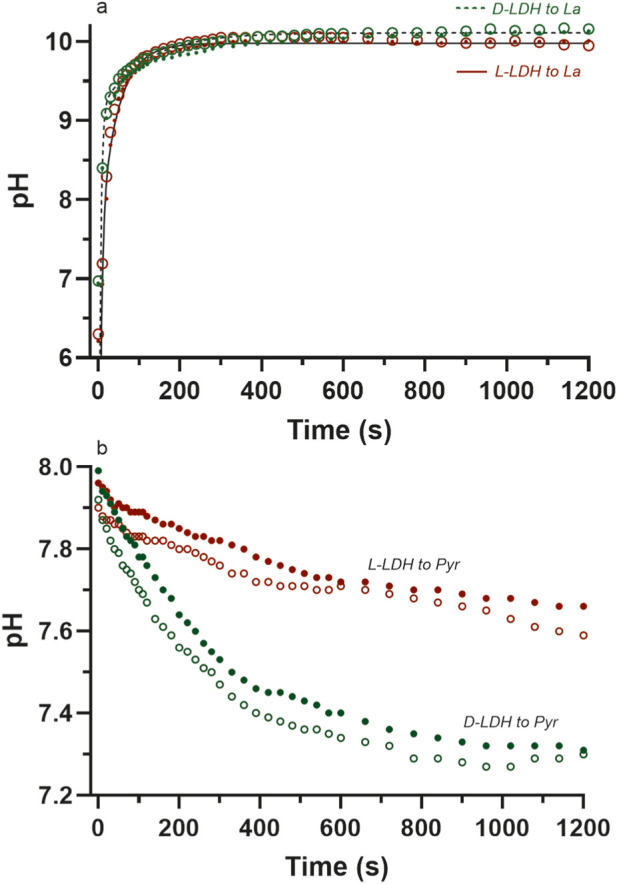
The pH data for replicate data collection for the pH time profiles of the L- (brown) and D-LDH enzymes (green) to each of **(a)** lactate and **(b)** pyruvate production. Data labels are also provided for visual clarity of the different data sets **(a)** to La: L-LDHa = • = LL6^tr^; L-LDHb = o = LL7^tr^; D-LDHa = • = DL6^tr^, D-LDHb = o = DL7^tr^; **(b)** to Pyr: L-LDHa = • = LP5^tr^; L-LDHb = o = LP6^tr^; D-LDHa = • = DP4^tr^; D-LDHb = o = DP5^tr^. See Table 2 for the pertinent data sets indicated by code and symbol (e.g., LL6^tr^).

**FIGURE 4:**
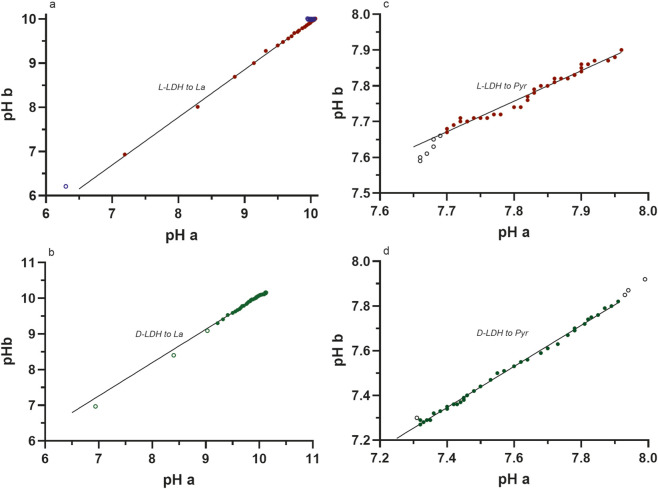
The graphic results of the linear regressions of the paired pH data for each L- and D-LDH enzyme and reaction direction. The individual graphs are for data for **(a)** L-LDH to lactate, **(b)** D-LDH to lactate, **(c)** L-LDH to pyruvate, and **(d)** D-LDH to pyruvate. Note the relatively narrow pH range of the data for the direction to pyruvate. As explained in Methods, the open circles represent the data that had to be removed from the linear regression analyses due to the relatively slow response of the pH meter (for the initial 30 s of the reaction to lactate) and for the pH profile conditions that caused stable pH data after about 10 min (this was mostly confined to the L-LDH reaction to lactate (blue open circles); see Discussion) (Table 4).

The test re-test reliability and validity was performed using commercial graphics and statistical software (Prism, GraphPad Software, Boston, MA, United States), where the regression correlation (reliability coefficient) between the data of two repeated trials for a given enzyme and specific reagent conditions was used to quantify reliability, and the standard error of the estimate (square root of the mean square error of the residuals) was used to quantify validity (lower residuals error = high validity). Not all data points (n = 42) were used for each linear regression of each reaction. This was due to the need to remove the initial data points (2–6 depending on the reaction) where the pH change was rapid for the L-LDH and D-LDH reactions to lactate production and clearly revealed the poor temporal resolution of the pH meter compared to the rapidity of the pH changes during the initial 30 s of these reactions. The end data points of each reaction also needed to be screened and removed after the stable peak pH had been obtained. Such procedures will be become more-clear from the data (Figures 3, 4; Table 3) presented in the Results section.

The computation of the expected change in the pH of pure water when H^+^ are added or removed (the equivalent of adding a very strong acid or base with complete dissociation and association, respectively) involved programming for Equations 3–10 in a commercial spreadsheet program (Excel, Microsoft Corporation, Redmond, WA, United States). Calculations were then completed for changes in pH for 0.0000025 M increments in product formation between 0.000001 and 0.001 M for both directions of the LDH reaction. Data were then transferred to a commercial graphics and curve fitting program as previously explained, where a two-function exponential association was applied for pH results of the LDH reaction to lactate (alkalosis), and a two-function exponential decay was applied for the direction to pyruvate (acidosis).

## 3 Results

The pH of deionized water for multiple samples per session, acquired across 14 laboratory sessions (n = 30) had a range of 5.08–6.85, with a mean ± SD of 6.02 ± 0.52. The [Substrates], enzyme optical isomer, enzyme activity and pH conditions (initial, start and end) for the pH temporal profiling of the different LDH reaction conditions are presented in Table 2. Select pH profiles of the initial 2 min baseline condition, followed by the L-LDH reaction to lactate and to pyruvate are presented in Figure 2. The baseline pH of the reagents was remarkably stable in contrast to the rapidity of the pH change that occurred after the enzyme was added.

The data for the test re-test reliability of the method of measuring the pH profiles of the chemical reactions, in addition to the chemical reactions themselves, is presented in Table 3 and Figures 3, 4. Note the very high squared reliability coefficients and the very low S_y.x_ results. The reactions, when equated for the same [S], _start_pH and enzyme activity, yield pH responses that are highly valid and reproducible.

**FIGURE 5:**
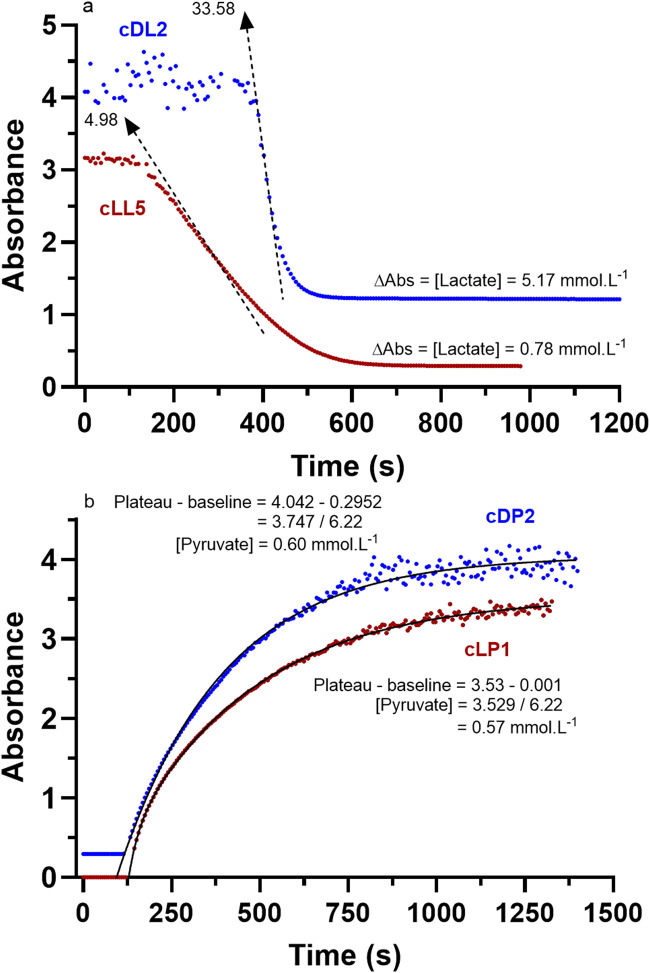
Select representative raw absorbance data (340 nm) from a subset of the cuvette reaction assays of Table 3 for **(a)** lactate production, and **(b)** pyruvate production. Note the labelled data for D- and L-LDH, and the final [Product] calculations. For **(a)**, note the absorbance variability and its elongated period of absorbance stability after the start of the reaction, where time is required to cause the decrease in NADH necessary to be detected in the functional range (0 to ∼3.2 units) of the spectrophotometer.

Figure 5 presents example raw data for absorbance using the cuvette method for each of lactate and pyruvate production for both D- and L-LDH. Note that the maximal absorbance for reliable data was not 4 units as stated in Methods, but 3.2 units. As such, the method was only suitable to verify the product formation from the pH profile conditions for the LDH reaction to pyruvate. Regardless, the data show the higher catalytic activity of the D-LDH and the constrained [Product] from the direction to pyruvate production for both LDH optical isomers.

**FIGURE 6:**
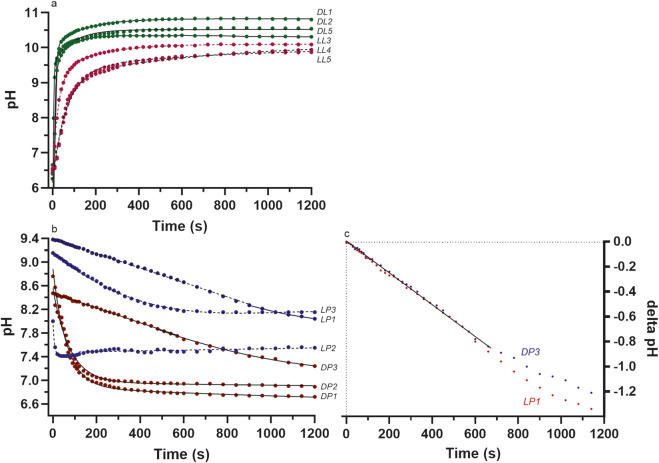
The pH data (baseline data removed) for different conditions of enzyme activity (U) and substrate concentrations ([S] for either lactate or pyruvate for **(a)** LDH to lactate production; **(b)** D- and L-LDH to pyruvate production. Note the near identical pH profiles for the D- and L-LDH isomers for pyruvate production when the different pH_start_ conditions are accounted for; **(c)** The similar profiles of LDH to pyruvate for the D- and L-LDH isomers when the pH data are expressed as delta (Δ) values from pH_start_. See Table 2 for more complete details of the [S] and enzyme U of each reaction.

The data of Figure 6 present further select conditions studied (D- and L-LDH for different enzyme activities and [Substrates]) from the pH profile analyses presented in Table 2. The LDH reaction directly altered the pH of the unbuffered *in-vitro* reagent, which adhered to the organic and computational chemistry of the chemical reaction to raise pH in the direction of lactate production, and lower pH in the direction of pyruvate production.

**FIGURE 7:**
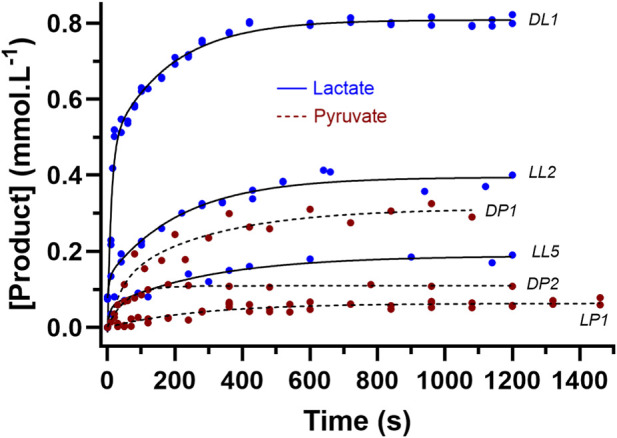
Select data sets of the product formation [Lactate] and [Pyruvate] data from L-LDH and D-LDH for the test-tube reagent method. See Table 2 for reaction code details.

For Figure 6a, note the resulting differences in the pH profiles of the varied reaction conditions were more dependent on the enzyme activity and type of optical isomer, not [Substrates]. This becomes understandable based on the small increases in [Product] (<1 mmol·L^-1^ total accumulated product formation over 20 min) (Figure 7). Nevertheless, the small [Product] over these profiles, and the plateau effects for pH suggest either pH itself was influential to enzyme activity, or there were added constraints to enzyme catalysis as the change in pH increased, or decreased, depending on the direction of the reaction (this is clarified later in Results). Regardless, note the blunted changes in pH for pyruvate production with reduced enzyme activity and [Substrates] compared to lactate production, and where such reductions were greater for L-LDH than D-LDH.

The LDH to lactate catalysis pH-time profiles (after correction for the different _start_pH values) were near visually identical between the D- and L-LDH optical isomers, though D-LDH had far greater catalysis (5-fold) for a given enzyme unit addition (Figure 6b) (see Discussion). The pH profile differences between the D- and L-LDH isomers were more notable for the LDH reaction to pyruvate production (Figure 6c), though the LDH reaction in this direction is more complicated based on the unfavorable bioenergetics (ΔG’° = +25.1 kJ⋅M^-1^) (20) and the varied influence of pyruvate and lactate inhibition specific to the LDH isozyme structure (H- vs. M-domain expression) (26–28, 30–35) (see Introduction and Discussion).

The increased [Product] was verified by reagent sampling and enzymatic assay of altered [NADH] or [NAD^+^] via enzyme spectrophotometry (Figure 7). These results were further supported by repeated reactions conducted in cuvettes involving the direct measurement of the changes in [NADH] via enzyme spectrophotometry (Table 4). Example raw data for absorbance from the cuvette method for each of lactate and pyruvate production for both D- and L-LDH were presented in Figure 5.

**TABLE 4:** Details of the cuvette assay method and conditions of the LDH reactions.

#	Direction→	Substrates*	Amount (g; mL)	mM⋅L^-1^^	Enzyme (U)	Peak [P]*
cLL1	Lactate	Na^+^-Pyruvate	0.0018	3.27	L-LDH: 5.0	3.3
		Na^+^-NADH	0.0118	3.33		
		Glycine^∼^	1.6 mL			
cLL2	Lactate	Na^+^-Pyruvate	0.0018	3.27	L-LDH: 1.0	3.23
		Na^+^-NADH	0.0118	3.33		
		Glycine^∼^	1.6 mL			
cLL3	Lactate	Na^+^-Pyruvate	0.0011	1.96	L-LDH: 1.0	1.92
		Na^+^-NADH	0.0071	2.0		
		Glycine^∼^	0.96 mL			
cLL4	Lactate	Na^+^-Pyruvate	0.0011	1.96	L-LDH: 0.4	1.90
		Na^+^-NADH	0.0071	2.0		
		Glycine^∼^	1.6 mL			
cLL5	Lactate	Na^+^-Pyruvate	0.0005	0.82	L-LDH: 0.2	0.78
		Na^+^-NADH	0.0030	0.83		
		Glycine^∼^	0.4 mL			
cLL6	Lactate	Na^+^-Pyruvate	0.0022	3.88	L-LDH: 2.0	0.80
		Na^+^-NADH	0.0027	0.83		
		Glycine^∼^	1.6 mL			
cLL7	Lactate	Na^+^-Pyruvate	0.0022	3.88	L-LDH: 0.5	0.79
		Na^+^-NADH	0.0027	0.83		
		Glycine^∼^	1.6 mL			
cLL8	Lactate	Na^+^-Pyruvate	0.0022	3.88	L-LDH: 0.2	0.78
		Na^+^-NADH	0.0027	0.83		
		Glycine^∼^	1.6 mL			
cLL9	Lactate	Na^+^-Pyruvate	0.0154	2.72	L-LDH: 0.35	0.53
		Na^+^-NADH	0.0019	0.58		
		Glycine^∼^	1.12 mL			
cDL1	Lactate	Na^+^-Pyruvate	0.0133	24.2	D-LDH: 1.2	5.26
		Na^+^-NADH	0.0193	5.4		
		Glycine^∼^	1.6 mL	32.0		
cDL2	Lactate	Na^+^-Pyruvate	0.0133	24.2	D-LDH: 0.8	5.17
		Na^+^-NADH	0.0193	5.4		
		Glycine^∼^	1.6 mL	32.0		
cDL3	Lactate	Na^+^-Pyruvate	0.0016	2.91	D-LDH: 0.1	2.81
		Na^+^-NADH	0.0107	3.02		
		Glycine^∼^	1.6 mL	32.0		
cDL4	Lactate	Na^+^-Pyruvate	0.0016	2.91	D-LDH: 0.4	2.78
		Na^+^-NADH	0.0107	3.02		
		Glycine^∼^	1.6 mL	32.0		
cDL5	Lactate	Na^+^-Pyruvate	0.0016	2.91	D-LDH: 0.25	2.63
		Na^+^-NADH	0.0107	3.02		
		Glycine^∼^	1.6 mL	32.0		
cLP1	Pyruvate	Na^+^-L-Lactate	0.003	5.36	L-LDH: 5	0.57
		Na^+^-NAD^+^	0.0171	5.21		
		Glycine^∼^	1.6 mL	32.0		
		Hydrazine^∼^	150 μL	30.0		
cLP2	Pyruvate	Na^+^-L-Lactate	0.003	5.36	L-LDH: 2.5	0.45
		Na^+^-NAD^+^	0.0171	5.21		
		Glycine^∼^	1.6 mL	32.0		
		Hydrazine^∼^	150 μL	30.0		
cLP3	Pyruvate	Na^+^-L-Lactate	0.003	5.36	L-LDH: 1	0.3
		Na^+^-NAD^+^	0.0171	5.21		
		Glycine^∼^	1.6 mL	32.0		
		Hydrazine^∼^	150 μL	30.0		
cLP4	Pyruvate	Na^+^-L-Lactate	0.0015	2.68	L-LDH: 2	0.21
		Na^+^-NAD^+^	0.0085	2.60		
		Glycine^∼^	0.8 mL	16.0		
		Hydrazine^∼^	75 μL	15.0		
cDP1	Pyruvate	Na^+^-D-Lactate	0.0028	5.0	D-LDH: 0.05	0.2
		Na^+^-NAD^+^	0.0169	5.1		
		Glycine^∼^	1.6 mL	32.0		
		Hydrazine^∼^	150 μL	30.0		
cDP2	Pyruvate	Na^+^-D-Lactate	0.002	3.6	D-LDH: 1.4	0.60
		Na^+^-NAD^+^	0.099	3.0		
		Glycine^∼^	1.6 mL	32.0		
		Hydrazine^∼^	150 μL	60.0		
cDP3	Pyruvate	Na^+^-D-Lactate	0.002	3.6	D-LDH: 1.25	0.58
		Na^+^-NAD^+^	0.099	3.0		
		Glycine^∼^	1.6 mL	32.0		
		Hydrazine^∼^	300 μL	60.0		
cDP4	Pyruvate	Na^+^-D-Lactate	0.002	3.6	D-LDH: 1.0	0.56
		Na^+^-NAD^+^	0.099	3.0		
		Glycine^∼^	1.6 mL	32.0		
		Hydrazine^∼^	300 μL	60.0		
cDP5	Pyruvate	Na^+^-D-Lactate	0.002	3.6	D-LDH: 0.5	0.37
		Na^+^-NAD^+^	0.099	3.0		
		Glycine^∼^	1.6 mL	32.0		
		Hydrazine^∼^	300 μL	60.0		

all reagent volumes were 5 mL *peak [Product] for the 20 min, mmol·L^-1^; ^adjusted for acid/base addition; ^∼^Glycine = 1 M stock, Hydrazine = 5 M stock.

**FIGURE 8:**
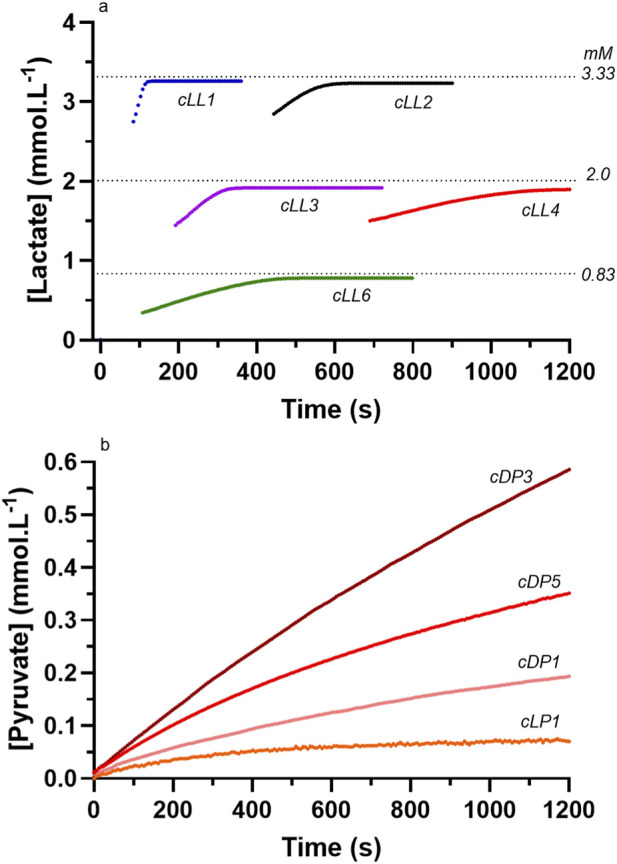
Select data sets of the product formation [Lactate] and [Pyruvate] data from L-LDH and D-LDH for **(a)** the [Lactate] data from the cuvette method, and **(b)** the [Pyruvate] data from the cuvette method. See Table 4 for reaction code details.

As documented for both lactate and pyruvate production, manual assay (Figure 7) and cuvette assay (Figures 8a,b) produced similar results for product concentrations for pyruvate and thereby provided confidence to explore the relationships between pH and [Product] under the different reaction conditions. Nevertheless, the [Product] data for the LDH reaction to pyruvate via the cuvette method resulted in much higher product formation. This verified the likely partial pyruvate inhibition of LDH in the reagent during the chemical reaction pH profiling experiments (see Discussion). In addition, as previously explained, the product formation for lactate for both D- and L-LDH via the cuvette method documented near completion of the reaction across all [Substrates] and enzyme activity conditions. This was expected based on the highly exergonic nature of this reaction direction, and as such reveals the pH-induced constraint to the chemical reaction during the unbuffered pH profiling of the reaction.

The data in Figures 7, 8 are interesting on multiple issues. For Figure 7, the variability in the absorbance for numerous samples assayed by traditional enzyme spectrophotometry was likely to be largely caused by sampling error of the reagent from the beaker during the pH profile data collections. Sampling 100 – 200 μL from 40 mL in time intervals of 10 s is possibly confounded by the incomplete distribution of product throughout the reagent within this time interval. As such, aberrant data points greater than 50% of a neighboring data point and deviant from the non-linear response profile (2 phase exponential association) were removed from the non-linear curve fitting. The low product concentrations for samples of lower [Substrates] and enzyme activity could not be assayed with sufficient sensitivity. This explained the greater reliance on the D-LDH data for the presentation of [Products], as these conditions resulted in larger [Products] which allowed for greater precision in the subsequent assays. For example, for the sampling and assay conditions employed in this research, a 0.1 mmol⋅L^-1^ change in product equated to a change in absorbance of 0.0061. To improve sensitivity of both the lactate and pyruvate assays, the duplicate sample volume was increased from 20 to 90 μL, increasing the absorbance of 0.1 mmol⋅L^-1^ lactate to 0.0257.

**FIGURE 9:**
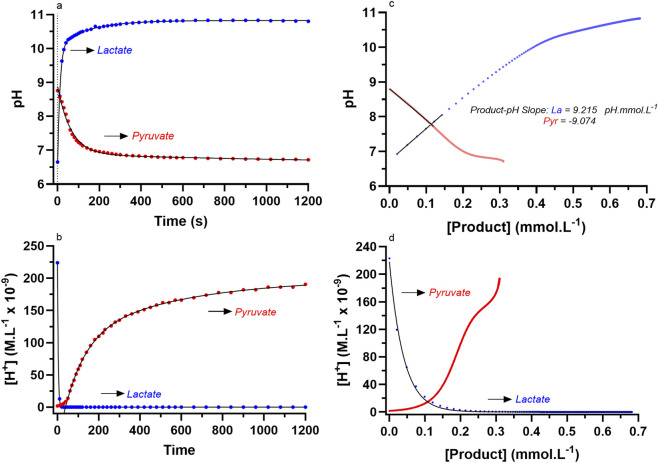
The data for the D-LDH reaction to lactate (DL1) and pyruvate (DP1) for **(a)** pH vs. time, **(b)** [H^+^] (_a_H_3_O^+^) vs. time, **(c)** pH vs. [Product], where the linear segment is identified by the solid back line of each data set, and **(d)** [H^+^] (_a_H_3_O^+^) vs. [Product].

**FIGURE 10:**
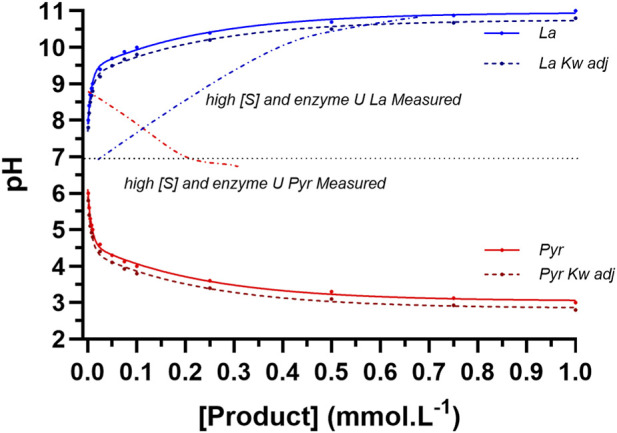
Data from the computation of expected pH profiles to product formation for the LDH reaction in the direction of each of pyruvate and lactate in pure water at 25 °C. Data are presented for unadjusted and adjusted values based on the inclusion of the ion product of water (Kw) corrected for ionic strength and do not account for the H^+^ buffering potential of the presence of pyruvate (Pyr), lactate (La), or the LDH enzyme in the reagent. The data sets from Figure 9 for lactate (La; DL1) and pyruvate (Pyr; DP1) production pH vs. [Product] profiles are included for comparison.

**FIGURE 11:**
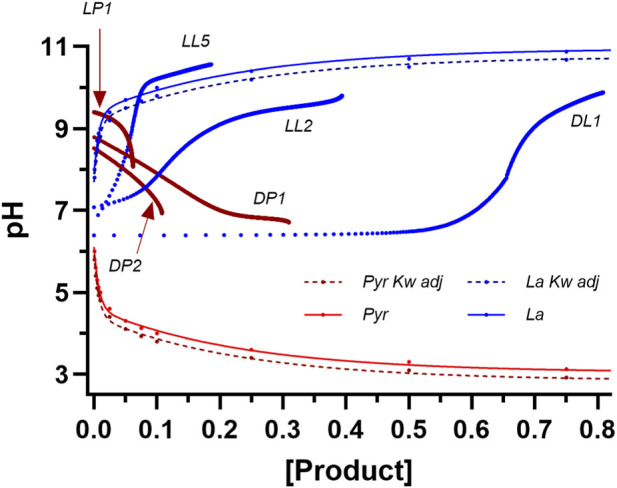
Data from the computation of expected pH profiles to product formation for the LDH reaction in the direction of each of pyruvate and lactate as per Figure 10. The additional data are for reaction specific pH and [Product] result curves (see Table 2 for coded reaction conditions). Note that for each of the pH to lactate production curves, the pH data commenced with a sigmoidal (highly buffered?) response, and more so for reaction conditions with larger [Product]. H^+^ buffering was less prominent in the direction of pyruvate production, regardless of the D- or L-LDH isozyme.

As shown in Figure 7, a clear non-linear profile (2 phase exponential association) was apparent for the assayed samples for both lactate and pyruvate. Though, note the smaller product formation and lower range of pH changes in the direction to pyruvate. The added aspect to factor into the data analysis is the role of the large pH changes in the unbuffered reagent, though as discussed below, there is evidence that the substrates, products and the enzyme provided some H^+^ buffering, in both directions of release and uptake (Figures 9–11).

As previously explained, the cuvette method was used to provide further evidence of the product formation of the reaction, which in turn supported the interpretation of the product formation rates and magnitude obtained from the traditional enzymatic assay of the reagent samples from the pH profile acquisitions. The cuvette method required a H^+^ buffer for enzyme activity and product formation. Such a different reagent condition resulted in a more rapid rate of product formation when sufficient enzyme activity was present (Table 4). The results of Figure 8 document the near completion of the cuvette-based reactions for LDH to lactate as expected due to the favorable energetics of this direction. The results for [Pyruvate] for the reverse direction of the reaction are larger than the traditional sample derived enzyme spectrophotometry method, which is to be expected as the cuvette method reagent contained hydrazine to remove production inhibition from pyruvate production. Regardless, the [pyruvate] results of Figure 8 reveal that the magnitude of the [product] for either of lactate or pyruvate are reasonable, and thereby infer some form of pH induced decrease in LDH catalytic function in the direction of lactate production.

Figure 9 presents the added original findings for comparing the pH temporal profiles of two examples of the LDH reaction to lactate and pyruvate, to their [P] to pH profiles. The most notable feature of this data is the similar pH to [Product] slopes (when accounting for the directional differences for the initial segment) for the initial segment of the data in both directions of the LDH reaction, where as documented in the figure, lactate production caused a pH:[Lactate] slope of 9.215 pH units.mmol.L^-1^, and a pH:[Pyruvate] slope of −9.074 pH units.mmol.L^-1^. This result occurred despite the differences in the magnitude and rate of product formation, and the direction, magnitude and rate of the pH ([H^+^] or _a_H_3_O^+^) changes. It is worth noting that these two reaction conditions represented those with the highest [Substrates] and enzyme activity levels (Figure 9c; see Figure legend, and Table 2). Thus, there is consistency in the H^+^_e_ in this reaction, in both directions, regardless of whether the H^+^ is a substrate or a product. This response is to be expected based on the hypothesis that the H^+^ involved in covalent modification of chemical reactions directly alters the pH of biological solutions. As such, the covalent H^+^_e_ in chemical reactions is likely to have direct biological relevance to cellular acid-base balance, and requires further investigation (see Discussion).

Despite the comments above, there remains concern that the pH to [Product] profiles of Figure 9c are influenced by factors other than the H^+^_e_ of the chemical reactions. Such factors could include the H^+^ buffering from the [Substrates], [Product] and enzyme in the reagent solutions, as yet unknown complexities of the chemical reaction pH response across a wide range of pH, and to whether such responses are different when confined to the more-narrow range of cellular acid-base conditions. In addition, are the pH to [Product] profiles of Figure 9c directly due to H^+^_e_ when considering that such H^+^_e_ in these chemical reactions is unitary, or stoichiometric for each product formed? Why are there differences in this pH:[Product] slope for different iterations of the pH temporal response of the LDH reactions (Figures 10, 11). Shouldn’t such unitary changes in H^+^_e_ cause a constant change to the pH:[Product] profiles in an unbuffered system? Would this response be the same for different chemical reactions that involve H^+^_e_? Can the expected pH changes from the H^+^_e_ in chemical reactions be calculated? How would the buffering provided by the [Substrates], [Product] and enzyme impact these profiles? What would be the influence of the ionic strength of the reagent to the pH response and product formation of each chemical reaction?

To investigate this further, calculations of the expected pH changes (in pure water) were completed as explained in Methods. The logic for this from solely a perspective of the computation of H^+^_e_ is that the LDH reaction to pyruvate would essentially involve the sequential addition of a strong acid (even though, as stated previously, this does not occur) causing complete dissociation. For the lactate direction, the reaction would be equivalent to the removal of a H^+^ stoichiometric to lactate production. The results for the computations of changes in pH for the LDH reaction to pyruvate and to lactate ranging in [Product] from 0.00001 to 0.001 M in increments of 0.0000025 M, for unadjusted and adjusted Kw, are presented in Figure 10. Such results reveal the calculated expected values for pH from the product formation of each direction of the LDH reaction if the reaction occurred in pure water (I = 0) and therefore no H^+^ buffering. As such, the computational data presented provided a reference comparison to experimentally acquired data from the perspectives of reaction direction, magnitude and non-linear profile.

The non-linear curve fitting is for two function exponential association and decay for lactate and pyruvate, respectively. Note that for the computational method, the reference (initial) pH of the pure water was assumed to be 7.0, which differs from the starting pH of the LDH reaction to pyruvate production. The previously presented lactate and pyruvate pH vs. [Product] profiles for the data of Figure 9c are provided for comparison. The initial interpretation of the data is that the theoretical curves reveal pH changes that are very similar to the measured pH vs. time profile data of the LDH reaction in either direction. Conversely, the pH vs. [P] profiles differed from the theoretically expected results. What could explain these differences?

Given that the chemical reactions were balanced by atoms, charge and ionic strength, the only possible explanation for a deviation in the pH to [Product] profiles would be H^+^ buffering from the reagent, which could only be caused by the [Substrates], [Product] and enzyme molecules of the reagent. To simplistically assess this, added pH profile data acquisitions were completed (see Methods) at lower [Substrates] and enzyme activity, along with reagent sampling and subsequent [Product] assay. The samples from prior LDH to lactate and LDH to pyruvate pH profile experiments of lower [Substrates] and enzyme activity were also assayed to compare pH to [Product] responses. The [Product] results of these added experiments were presented in Figure 7, and the resulting pH vs. [P] profiles in comparison to the computed data are presented in Figure 11.

The three curves presented for lactate production in Figure 11 show responses consistent with H^+^ buffering, with the results of the D-LDH 10 U, 10 mM condition being the most obvious in the blunted pH response for increased product (lactate) formation. It is worth noting that the reason for the extended delay in the pH response in this [Product] x-axis figure presentation is because for most of the D-LDH reactions to lactate production, 80% of the pH response was complete within the initial 1 min of the reaction (see Figure 6a), and it is difficult to accurately profile product formation during this short time period.

The difficulty with the experimentation of this topic is the need for more [Substrates] and/or enzyme activity to foster more product formation. However, each of these adjustments lead to increased H^+^ buffering by the enzyme, and changes in [Substrates] and [Product]. Efforts to reduce the H^+^ buffering in the reagent through lowered [Substrates] and enzyme activity lower the magnitude of [Product]. This is clearly seen in Figure 11 for the L-LDH reactions for both lactate and pyruvate production. The consistently lower [Products] for both D- and L-LDH for pyruvate production is likely indicative of the product inhibition from pyruvate. Less product means a reduced sensitivity of the method and assays of product formation. This is especially problematic when most of the product formation occurs in the initial 30 s to 1 min of the chemical reaction. Lowering enzyme activity further to extend the time-period of product formation is counter-productive due to the lower magnitude of product formation.

## 4 Discussion

The purpose of this research was to document the direct influence of the H^+^_e_ of the D- and L-LDH reactions, in both directions, to the pH of an *in vitro* reagent. All 5 items of the purposes of this research were answered. 1. The LDH reaction, for both D- and L-LDH, produces lactate or pyruvate, depending on the direction of the reaction, and not their acidic structures. 2. The H^+^_e_ of the LDH reactions functions as indicated by the organic chemistry and resultant computational chemistry, where lactate production increases pH and pyruvate production decreases pH. 3. Such pH changes were persistent across 20 min of the chemical reactions, are highly reproducible, and further influenced by enzyme activity, [S] and subsequent product accumulation. 4. The prior results confirm that the LDH reaction H^+^_e_ is consistent with prior computational chemistry evidence. 5. There is potential for developing a computational strategy for such chemical reaction H^+^_e_, though considerably more research is needed to more accurately quantify this chemical behavior and better document the determinants to such pH changes.

The most important and impactful results of this research concern the pH changes to the reagent that occur during the H^+^_e_ of the LDH reaction. Such results present evidence that opposes the traditionally accepted organic acid origin of metabolic pH disturbances. While the methods are relatively simple, the logical task of testing the pH changes for given chemical reactions has been an overlooked feature of acid-base biological chemistry to current time. Perhaps this speaks to the dominance (since the late 19th century) of the traditional understandings of acid-base chemistry and biochemistry as previously mentioned and more thoroughly detailed in the Introduction. While details of the kinetics of the LDH reaction are also presented for different enzyme optical isomers, enzyme activity and [S], such added experimental inquiry was completed to explore the unknown optimal conditions needed to profile the consequent pH changes across a sufficient timeframe, with quantifiable product formation.

The results of Figures 5–7 and Tables 1, 2 prove that pyruvate conversion to lactate is H^+^ consuming, lactate conversion to pyruvate is H^+^ releasing, and more importantly, that the H^+^_e_ involved in these chemical reactions alters the pH of the *in-vitro* reagent. As proposed based on the reaction mechanism of LDH being identical for the D- and L- optimal isomers of LDH, the pH profile results were almost identical for D- and L-LDH reactions in the direction of lactate production. In the direction of pyruvate production, pH profiles and [Product] were both lower than for lactate production, with less activity for L-LDH than D-LDH. Consequently, the results provide additional proof to the evidence from computational chemistry that the LDH reaction does not produce lactic acid or pyruvic acid, but instead their ionic structures, lactate and pyruvate.

The data of Table 3 and Figures 3, 4 document the impressive test re-test reliability of the methods and also the H^+^_e_ of the LDH reactions of this research, in either direction of product formation. This is especially true for the direction to lactate production. The reduced activity of the LDH direction to pyruvate, combined with the role of pyruvate as an LDH inhibitor, explains the relatively lower reproducibility of this reaction, though still of high statistical significance (Table 3). Nevertheless, in the direction of pyruvate production, pH profiles and [Product] were both lower than for lactate production, with less activity for L-LDH than D-LDH despite the intentionally higher added enzyme activity for L-LDH. The greater rates and magnitude of product formation for the D-LDH isozyme is noteworthy given that the L-LDH and D-LDH used in this research were from different cell sources (Table 1) and were likely to have different H- vs. M- domain proportionality. In being bacterial and therefore more glycolytically dependent on ATP turnover, the D-LDH would be hypothesized to be predominantly M-domain structured (see Introduction). We were unable to retrieve research of the structural molecular biology of the two LDH isozymes used in this research.

Given that this research was of two balanced chemical reactions (for atoms and charge) further reveals that the only causal mechanism for altering pH was the direct H^+^_e_ based on the H^+^ acting as either a substrate or a product of the chemical reaction, depending on the direction (which metabolite is the main substrate or product) of the reaction. The pH profile results of the LDH reaction align with the computational chemistry results of Robergs (2017), where the pH specific H^+^ coefficients of the LDH reaction for pH = 6 vs. 7 were documented to be 1.0044 vs. 1.0004, respectively. Application of the methods of Robergs (2017) for the reversal of the LDH reaction to pyruvate production reveal pH specific H^+^ coefficients for pH = 6 vs. 7 to be −1.0044 vs. −1.0004, respectively. Note that these H^+^ coefficients are not +1 and −1, respectively, because they are slightly modified by inclusion of the differences in pH dependent H^+^ association between the substrates and products of the chemical reaction, which are due to the differences in the dissociation constants of these molecules. Such pH dependent H^+^ association/dissociation features of specific chemical reactions (oxidation-reduction, phosphate transfer, and hydrolysis reactions) are not accounted for in the text explanations of the chemical structures, or the text content of acid-base chemistry that are presented in textbooks of biochemistry (Voet and Voet, 2018; Nelson and Cox, 2021).

We recognize the limitations of the isolated chemical reactions in an *in vitro* reagent. However, the chemistry of the LDH reaction in a beaker should be similar to what occurs in a cell; the substrates and products are the same, the reaction mechanism is the same, and so should be the expected H^+^_e_. The *in-vitro* nature of this research revealed large pH perturbations, which are of course non-physiological and may have also induced lowered enzyme activities. This is to be expected in a non-buffered (or minimally buffered) system. Nevertheless, such pH perturbations are important to recognize as they show the capacity of the LDH reaction to lactate or to pyruvate for directly altering pH, and such knowledge can only be gained from an unbuffered, and hence, *in-vitro* system. Such capacities are important for re-evaluating the buffering needed to retain pH values within the physiological range for the biological tissue of interest (cellular vs. systemic). There are also implications of the results of this research to better understand the metabolic determinants of H^+^ release and removal. As such evidence opposes traditional acid-base chemistry understanding, there is a need to re-evaluate the computation of the source and capacity of H^+^ during metabolic causes of acidosis and ascertain how this may affect the capacity of buffering systems *in-vivo*.

In a cell the bioenergetics of a chemical reaction may change based on altered [Substrates] and [Products]. For any given bioenergetic conditions, enzymes further function to alter the kinetics (rate) of the reaction while not interfering with the intrinsic reactivity of the chemical reaction that determines the standard free energy change (Equation 11, and see later Discussion content) (Voet and Voet, 2018; Nelson and Cox, 2021; Rumble, 2021). As mentioned in all textbooks of biochemistry, enzymes, as biological catalysts, play no role in altering the bioenergetics (Gibb’s free energy changes) and therefore the directionality of a chemical reaction (Voet and Voet, 2018; Nelson and Cox, 2021; Rumble, 2021). Based on these axioms of chemistry and physics, the results presented in Figures 2–8 reveal that the pH changes are driven by product formation, and hence the organic chemistry of the chemical reactions combined with the rate of the chemical reactions (enzyme regulation and [Substrates] and [Products]). When applied to cellular chemistry within biological systems, the multifaceted metabolic milieu of the cell governs the rates of product formation, the presence and concentration of H^+^ buffering compounds and therefore the net H^+^_e_. Consequently, this complexity of *in vivo* metabolic regulation and function must eventually be included in a multifactorial understanding (H^+^_e_ + water ionization + H^+^ buffering + strong ion differences + longer term renal compensations) of how a subset of chemical reactions, in addition to the functions of multiple physiological systems, might collectively alter cellular and systemic acid-base balance *in vivo*.

$$\Delta G=\Delta G^{{{}^{\circ}}^{\prime }}+\left(\left(\frac{R\;T}{1000}\right)\left(\ln\left(\frac{\left(\left[C\right]\;\left[D\right]\right)}{\left(\left[A\right]\;\left[B\right]\right)}\right)\right)\right)$$ (11)

where ΔG and $\Delta G^{{{}^{\circ}}^{\prime }}$ are expressed as kJ·M^-1^; R = 8.315 J·M^-1^·K^−1^; T = °K; [C], [D] = products; [A], B] = substrates

The ramifications of these results to acid-base chemistry applied to biological systems are important. The results conform to prior computational chemistry based on molecular dissociation constants (Robergs et al., 2004; Robergs, 2017; Robergs, 2019; Robergs, 2021; Kushmerick, 1997; Vinnakota et al., 2006) and directly challenge the long-held dogma of lactic acid being the main source of the H^+^ release in cells taxed by an excessive demand for ATP turnover, or the constrained cellular capacity for ATP regeneration. While carboxylic acids, such as lactate and pyruvate (and many other molecules) are structurally classified as acids, they are not acids in that they do not release a H^+^ to solution once produced in their negatively charged anion form. This means that there is a need to develop a mechanistic and computational understanding of cellular and systemic acidosis devoid of acids as the source of the H^+^. Such evidence may also impact the current understanding and application of the Stewart physico-chemical theory for the cause of systemic acidosis (Stewart, 1983). Core to Stewart’s computational approach is the assumption that no direct metabolic source exists for H^+^ release to the aqueous solution of a biological system; that the H^+^ activity of an aqueous solution is a totally ‘dependent’ variable. The results of Figures 1–7 present evidence that disprove this assumption. As such, Stewart’s computations may need adjustment for metabolic derived chemical reaction H^+^_e_, or possibly a new approach needs to be derived and proposed that encompasses multiple components that contribute to metabolic acidosis.

As mentioned, the applications of acid-base chemistry computation to biological systems may need a reevaluation of the origins of metabolic acidosis. The results support the view that when concerned with cellular metabolism, acidosis is likely to be caused (or contributed to) by a metabolic systems alteration where the contributions of the chemical reactions involved in cellular ATP turnover combine to cause net H^+^ release, which is capable of directly altering cellular and systemic pH. If the results of this research are consistent across many other chemical reactions of cellular metabolism, then no cellular metabolic organic acids are produced by the chemical reactions linked to increased ATP turnover. How this changes our understanding of the quantification and prediction of the H^+^ load of acidosis, and the capacity of the collective (and individual) systems that are/is needed to buffer it remains to be explored.

We have attempted to do this with Equations 1–8 for pertinent data presented in Figures 8–11. Simple alterations of the Henderson-Hasselbalch equation revealed the expected pH changes in pure water for known concentrations of direct H^+^ release or uptake with further adjustments to the ion product of water caused by a moderate ionic strength of the biological reagent. Further research now needs to be done to further challenge, refine and expand this computational approach with pH dependent H^+^ buffering, and long-term acid-base perturbations based on renal contributions to H^+^ and HCO_3_^−^ handling and strong ion adjustments.

Finally, there are comments to make regarding the lowered kinetics of the LDH reaction to pyruvate vs. lactate (Figures 2–9) and what this might mean to growing interest in the role of lactate entry into mitochondria for its oxidation to pyruvate (Brooks et al., 2022). The results from this research are clear. Despite chemical reactions that commenced with relatively high [Substrates] and no [Product], and hence highly favorable bioenergetics to [Product] formation, the LDH reaction in the direction of pyruvate were orders of magnitude lower in kinetics than in the direction of lactate. Such kinetics were even worse for the L-LDH vs. D-LDH optical isomer, and the results adhere to the highly exergonic bioenergetics of the LDH reaction to lactate production, where the standard Gibb’s free energy exchange (ΔG’°) equates to −25.1 kJ·M^-1^ (28), which can be altered based on the changeable *in-vivo* [Substrates], [Products] and temperature of a cell (Equation 11).

To enable the LDH reaction to proceed toward the formation of pyruvate rather than lactate, there needs to be more than a 15,000-fold increase in the mass action ratio of the reaction (([Lactate] [NAD^+^])/([Pyruvate] [NADH])) (computed in the direction of lactate production). By convention, the [H^+^] is not factored into the bioenergetic calculations. Such changes are unlikely to occur in living (biological) systems unless the product (pyruvate) is rapidly removed by other chemical reactions (Storey, 2016; Brooks et al., 2022). Pyruvate production was possible in this research due to the controlled start [Substrates] and [Product] ([Product] = 0 mmol·L^-1^) conditions of the LDH reactions. The constrained [P] formation in the direction of pyruvate production can be further explained by the rapidly diminishing free energy release of the reaction as [Pyruvate] and [NADH] increased and [Lactate] and [NAD^+^] decreased. Furthermore, such constraint is magnified by the known pyruvate inhibition of LDH in the direction of pyruvate production, which as previously explained, is more pronounced in LDH isozymes rich in H-type sub-units (Zewe and Fromm, 1962; Eggert et al., 2011; Wang, 1997; Powers et al., 2007; Latner et al., 1966; Storey, 2016; Gray et al., 2014; Callender and Dyer, 2015; Karlsson et al., 1974; Alberts et al., 2024).

Interestingly, given the results of this research, the [H^+^] would be influential in the LDH reaction given the now documented importance of the H^+^ as either a substrate or product of the reaction, depending on the direction of product formation. In addition, based on the chemiosmotic theory of oxidative phosphorylation (Alberts et al., 2024), and given the H^+^ release in the direction of pyruvate production, the high H^+^ activity of the aqueous inter-membranous space should oppose/retard the reduction of lactate to pyruvate in this specialized, subcellular, intra-organelle space. Interestingly, Kane (2014) proposed that the involvement of mitochondrial electron shuttle systems could minimize this complication. Yet at this time, the computational or biological relevance of these assertions are unknown. Consequently, the reality of the unfavorable bioenergetics of the LDH reaction in the direction of pyruvate production, in addition to the pyruvate inhibition of LDH, need to be better accounted for in postulates of mitochondrial LDH activity and its roles in the intramitochondrial oxidation of lactate to pyruvate.

## 5 Conclusion and recommendations

Experiments were conducted of the controlled activity of the LDH reaction (for each of L- and D- optimal isomers) in an *in vitro* biological reagent for a range of different [Substrates] and enzyme activities. No added H^+^ buffers were included in the reagent. The LDH reaction was investigated in each direction of lactate and pyruvate production. Results revealed similar pH profiles for D- and L-LDH reactions in the direction of lactate production, though less activity to pyruvate production and especially so for the L-LDH reaction. The test re-test reliability of both LDH isozymes, in either direction of lactate or pyruvate production, all had high reliability coefficients (all R^2^ > 0.97, and p < 0.0001). The results are clear in documenting that the LDH reaction directly alters the pH of the aqueous solution it exists within. Lactate production caused a rapid and large increase in pH, with moderate changes in [Lactate] approximating 0.4–0.9 mmol·L^-1^ ([Substrates] and enzyme activity dependent) over 20 min. Conversely, pyruvate production decreased pH, but with lower magnitude changes in both pH and [Pyruvate] (0.1–0.3 mmol·L^-1^) compared to lactate production. Based on the pH changes, it is clear that the LDH reaction produces lactate or pyruvate and not lactic acid or pyruvic acid, depending on the directionality of the reaction. The pH and [Products] results also verify that the H^+^_e_ in chemical reactions, as either a substrate or a product, are likely to have biological relevance and need to be accounted for in models of cellular H^+^_e_ causal to the development of cellular and systemic acidosis or alkalosis. As there are numerous other cellular reactions that involve H^+^ as a substrate or product, the results of the LDH reaction reveal that similar H^+^_e_ should also occur for these reactions.

This research was highly exploratory. No prior research has been done to quantify the influence of chemical reactions on the pH of an *in-vitro*, unbuffered reagent. Regardless, the results document the likely need for change in our understanding of cellular H^+^_e_ and how this biological chemistry contributes to cellular and systemic acidosis. Similarly, the results challenge the traditional/historic assumption that cells produce acid molecules, and it is these organic acids that cause acidosis. Similarly, as the results of this research show that the [H^+^] is not a dependent variable; chemical reaction H^+^_e_ directly alters the pH of an aqueous solution, there is a need to re-evaluate the proportional contributions of changes in strong ion concentrations to blood acid-base balance. As such, the computational structure of the Stewart approach may need to be altered accordingly.

Based on the results, more research is needed on added chemical reactions that involve H^+^ as a substrate or product, the inclusion of known H^+^ buffers, and the study of simultaneous multiple reactions and pathways to address the added complexity of cellular physiological systems. The different responses of the LDH reaction for known differences in the domain structure of the isozyme are also of importance to understanding the pH responses of the LDH reaction, and added research needs to occur on this issue. Eventually, once the basic science responses of H^+^_e_ of chemical reactions are better understood, methods should be developed to regulate specific reactions of cellular systems to ascertain the *in-vivo* responses and implications of targeted chemical reactions to cellular acid-base balance. Further cell systems should also evaluate the interaction between cytosolic chemical reaction H^+^_e_ and mitochondrial H^+^ handling. There is also logic to the open-minded re-development of a theory of acid-base balance that incorporates multiple determinants to the [H^+^] (aH_3_O^+^) of biological solutions. Such research directives are important for how they could collectively improve applications or interventions designed to cause adaptations to the metabolic systems involved in diminishing cellular and systemic acidotic perturbations, as well as improve the prevention and treatment of the acidosis linked to numerous disease processes. As stated by Warren and Hedrick (2020), the lack of understanding of the biochemical origins of lactate and proton release constrain the comprehension of acidosis in animals. Added attention must be directed to acid-base chemistry and physiology so that this life supporting process can be better understood for the benefit of all.

## Data Availability

The raw data supporting the conclusions of this article will be made available by the authors, without undue reservation.
